# First Detection of Porcine Hemagglutinating Encephalomyelitis Virus (PHEV) in Wild Boars (*Sus scrofa*) from Southern Italy: A Two-Year Epidemiological Survey

**DOI:** 10.3390/vetsci13080773

**Published:** 2026-08-02

**Authors:** Chiara Ortello, Viviana Manzulli, Lorenzo Pace, Antonella Cristina Romano, Mariateresa Toce, Lucia Palazzo, Donatella Farina, Laura Del Sambro, Valeria Rondinone, Luigina Serrecchia, Domenico Galante

**Affiliations:** 1 PhD National Programme in One Health Approaches to Infectious Diseases and Life Science Research, Department of Public Health, Experimental and Forensic Medicine, University of Pavia, 27100 Pavia, Italy; chiara.ortello01@universitadipavia.it; 2 Istituto Zooprofilattico Sperimentale della Puglia e della Basilicata, 71121 Foggia, Italy; lorenzo.pace@izspb.it (L.P.); antonella.romano@izspb.it (A.C.R.); mariateresa.toce@izspb.it (M.T.); lucia.palazzo@izspb.it (L.P.); donatella.farina@izspb.it (D.F.); laura.delsambro@izspb.it (L.D.S.); valeria.rondinone@izspb.it (V.R.); luigina.serrecchia@izspb.it (L.S.); domenico.galante@izspb.it (D.G.)

**Keywords:** coronaviruses, PHEV, wild boars, epidemiological survey

## Abstract

In recent years, the wild boar population has increased in Italy. Wild boar is one of the most problematic species from a sanitary point of view because it represents a dangerous reservoir of pathogens and viruses. The aim of this study was to investigate the circulation of coronaviruses in wild boars, a group of viruses known for their ability to spill over between species. As part of the wild boar population control plan established by the Basilicata region relating to this species, 625 animals were analyzed from May 2024 to January 2026. Nasal swabs were collected at the slaughterhouse in the province of Potenza. Screening was conducted using pan-coronavirus PCR through a nested PCR assay targeting the conserved RNA-dependent RNA polymerase (*RdRp*) region. Out of all samples analyzed, 1.9% were positive for Porcine Hemagglutinating Encephalomyelitis Virus (PHEV), marking the first identification of this neurotropic coronavirus in wild boars in Italy. The detection of PHEV in free-ranging populations highlights the potential for viral circulation at the wildlife–livestock interface and underlines the importance of integrated surveillance systems to prevent possible interspecies transmission and minimize the risk of spillover to domestic pig herds.

## Simple Summary

Wild boars are increasingly widespread in Italy and can act as reservoirs of infectious agents that threaten animal health. Among these agents are coronaviruses, which are known for their ability to infect different species. This study investigated the presence of coronaviruses in 625 wild boars from the Basilicata region between 2024 and 2026. Molecular analyses identified Porcine Hemagglutinating Encephalomyelitis Virus in 12 animals, representing the first report of this virus in wild boars in Italy. Although the prevalence was low, the finding suggests that wild boars may contribute to the maintenance and circulation of this coronavirus in the environment.

## 1. Introduction

Wild boars (*Sus scrofa*) are the largest mammals found in the wild worldwide, and over the last 30 years, their population has undergone a systematic increase in size and geographical distribution across Europe [1]. In recent years, Italy has also seen a steady growth in the wild boar (*Sus scrofa*) population, due to this species’ high adaptability [2]. For some Italian regions, the population density increased despite the high number of animals hunted [3]. The population increase has been associated with numerous economic, environmental, and social problems [4]. To date, interest in wild boars has considerably increased, as this species is susceptible to different zoonoses and diseases also affecting domestic pigs, thanks to its ability to adapt to urban and peri-urban environments, representing a potential direct and indirect risk to both animal and human health. For all these reasons, wildlife surveillance plans are becoming increasingly important [5]. Wild boars can be reservoirs for bacteria, viruses, and parasites, which are transmissible to humans and domestic animals through direct or indirect interactions [6]. Wild boars can transmit important infectious diseases to domestic animals, such as classical swine fever, brucellosis and trichinellosis, and to humans, including coronaviruses, hepatitis E, tuberculosis, leptospirosis and trichinellosis [7,8]. Coronaviruses are RNA viruses with large genomes, high mutation rates, and frequent recombination events, which facilitate rapid adaptation to new hosts and ecological niches [9]. In animals, they can cause respiratory, enteric, and neurological diseases. Their replication mechanisms and recombination potential increase the likelihood of cross-species transmission and enhance their zoonotic potential [10]. The emergence and evolution of coronaviruses in domestic pigs (*Sus scrofa domesticus*) and wild boars (*Sus scrofa*) represent a growing concern for animal and public health, particularly due to the risk of spillover into swine production systems [11,12]. Although human-to-human transmission of porcine coronaviruses has not been confirmed, interspecies transmission remains a relevant threat, especially in areas with high animal densities, limited surveillance, or poor sanitary conditions [13]. Swine are susceptible to several species of coronavirus, including Porcine Epidemic Diarrhea Virus (PEDV), Transmissible Gastroenteritis Virus (TGEV), Porcine Respiratory Coronavirus (PRCV), Porcine Deltacoronavirus (PDCoV), Porcine Acute Diarrhea Syndrome Coronavirus (SADS-CoV), and Hemagglutinating Encephalomyelitis Virus (PHEV) [14]. PHEV is particularly important from an economic perspective because it primarily affects piglets under three weeks of age. After entering through the respiratory or gastrointestinal tract, the virus can invade peripheral nerves and reach the central nervous system via the vagus and trigeminal nerves [15,16], often causing high mortality and substantial economic losses for farmers.

Despite the relevance of coronaviruses in domestic swine, the role of wild boars in their ecology and transmission remains poorly understood. This study aims to investigate the circulation of porcine coronaviruses—particularly respiratory ones—in wild boars to identify potential risks for both animal and human health.

## 2. Materials and Methods

### 2.1. Sample Collection and RNA Extraction

Following the wild boar culling plan implemented by the Basilicata region (Southern Italy), nasal swab samples were collected between May 2024 and January 2026 (Table S1) and stored at −20 °C until processing. For analysis, nasal samples were resuspended in 2 mL of sterile phosphate-buffered saline (PBS). Automated RNA extraction from 200 µL of nasal swabs was performed using the MagMAX™ Viral/Pathogen Nucleic Acid Isolation Kit (Thermo Fisher Scientific, Waltham, MA, USA) on the KingFisher Flex instrument (Thermo Fisher Scientific, Waltham, MA, USA), according to the manufacturer’s instructions. RNA was eluted in 80 μL of elution buffer.

### 2.2. Biomolecular Analysis

Two types of PCR, a one-step RT-PCR and a nested PCR, were used for sample analysis. Specifically, one-step RT-PCR involves the amplification of the *RdRp* gene that encodes the most conserved protein domain of α-, β-, γ-, and δ-CoVs, using primers described by Hu et al., 2018 [17]. Reagent concentration and thermal profile of PCR were the same as described by Xiu et al., 2020 [18]. For one-step RT-PCR, amplification was performed using the CFX96 Connect Real Time PCR Detection System (Bio-Rad, Hercules, CA, USA) as a conventional thermal cycler. No fluorescence data were acquired during amplification, and PCR products were analyzed by 2% agarose gel electrophoresis.

Each 25 μL reaction mixture contained 12.5 μL of 2× Reaction Mix (Invitrogen, Thermo Fisher SuperScript™ III Platinum™ One-Step qRT-PCR Kit, Waltham, MA, USA), 1 μL of each primer (25 μM), 0.5 μL Taq DNA Polymerase SuperScript™ III RT/Platinum™ (Thermo Fisher Scientific, Waltham, MA, USA), 5 μL RNA and nuclease-free water to the final volume. Thermal cycling was performed at 55 °C for 30 min for reverse transcription, followed by 94 °C for 2 min and then 35 cycles of 94 °C for 15 s, 54 °C for 30 s and 68 °C for 60 s. The amplicons obtained were subjected to nested PCR, using two internal primers of the *RdRp* sequence. The sequence of the primers used is shown in Table 1. For nested PCR, amplification was performed using the Thermal Cycler 2720 (Thermo Fisher Scientific, Waltham, MA, USA). Each 25 μL reaction mixture contained 2.5 μL of 1× PCR reaction buffer, 1.5 μL MgCl_2_ (25 mM), 0.5 μL dNTP (10 mM), 1 μL of each primer (0.4 μM), 0.25 μL Taq DNA Polymerase (QIAGEN GmbH, Hilden, Germany), 2 μL RNA and nuclease-free water to the final volume. Thermal cycling steps were 94 °C for 5 min for initial denaturation, followed by 35 cycles of 94 °C for 15 s, 54.5 °C for 30 s and 72 °C for 60 s. Expected amplicons were 670–673 bp and 559–602 bp respectively. Negative and positive controls were included. Amplification products were analyzed through 2% agarose electrophoresis at 120 V for 25 min. The gel visualization was performed by a Gel Doc XR+ System (Bio-Rad, Hercules, CA, USA).

Subsequently, the positive samples were subjected to two real-time RT-PCRs specific to respiratory coronaviruses, PHEV and PRCV, as the starting material used was nasal swabs. real-time RT-PCR was set up in a final volume of 25 μL, including 5 μL TaqMan™ Fast Virus 1-Step Master Mix for qPCR (Thermo Fisher Scientific, Waltham, MA, USA), 0.25 μL of each primer (0.5 μM) and 0.75 μL probe (0.3 μM), 5 μL RNA and nuclease-free water to the final volume. Thermal cycling steps included an initial denaturation at 50 °C for 10 min, a step at 95 °C for 3 min followed by 40 cycles at 94 °C for 10 s and a final step at 56 °C for 30 s [19,20]. The results were analyzed by the Bio-Rad software CFX Maestro 1.1 with baseline threshold setting at 100 RFU.

**Table 1:** List of one-step, nested-PCR and real-time RT-PCR primers and probes used in this study.

PCR Type	Primers/Probes	Sequences (5′-3′)	References
One-step	Forward	AARTTYTAYGGHGGYTGG	[17]
	Reverse	GARCARAATTCATGHGGDCC	
Nested	Forward Reverse	GGTTGGGACTATCCTAAGTGTGA CCATCATCAGATAGAATCATCAT	[18,21]
PRCV	Forward	TTGTCTGGGTTGCCAAGGAT	
	Reverse	CATCGAATYTCAAAGCTTTGGATT	[20]
	Probe	FAM-ACKCTTGGTAGTCGTGG-BHQ1	
PHEV	Forward	CCAGAAGGATGTTTATGAATTGC	
	Reverse	CCTGATGTTGATAGGCATTCA	[19]
	Probe	FAM-TGGCGCGATTAGATTTGAYAGCACACTC-BHQ1	

### 2.3. Data Analysis

Descriptive statistical analyses were conducted to determine the frequency and proportion of PHEV-positive samples based on the 95% confidence interval (CI) diagnostic test. The corresponding 95% confidence interval (CI) was estimated using the Wilson score method for binomial proportions [22].

## 3. Results

**Figure 1:**
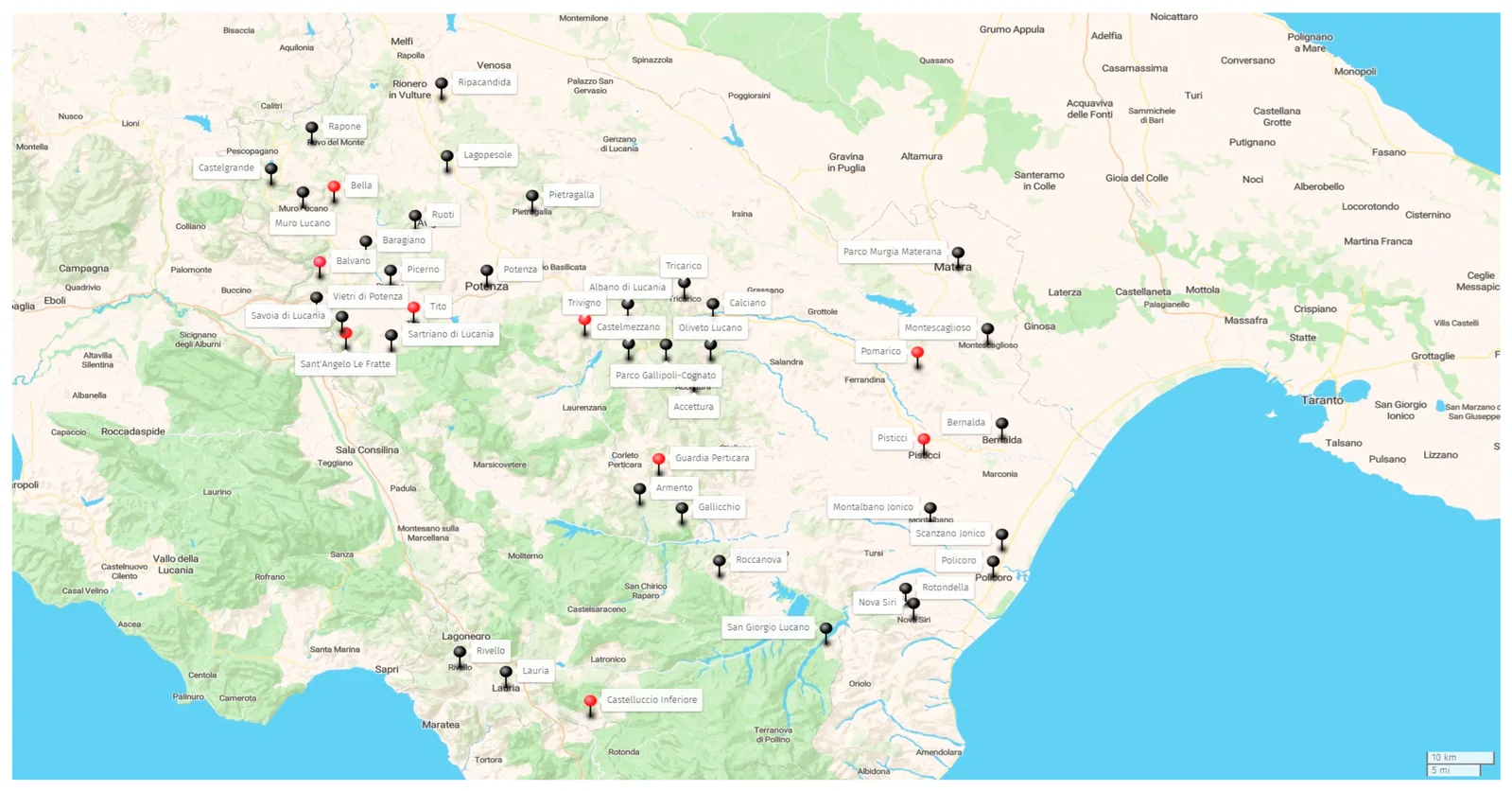
Geographic distribution of sampling sites in the Basilicata region (Southern Italy). The map shows PHEV-positive samples in red and PHEV-negative samples in black.

In this study, a total of 625 animal samples (Figure 1) were initially analyzed with the pan-coronavirus assay, which includes two types of PCR: a one-step RT-PCR and a nested PCR. Following the initial screening, 12 positive samples were identified. Subsequently, these positive samples were subjected to two real-time RT-PCR assays targeting respiratory coronaviruses, PHEV and PRCV. All 12 samples tested positive for PHEV while testing negative for PRCV. Descriptive statistical analyses were performed to summarize the study data. Overall, 12 out of 625 wild boars tested positive for PHEV, corresponding to an prevalence of 1.9% (95%CI: 1.11–3.33%), calculated by the Wilson score method. The cycle threshold (Ct) values obtained by PHEV real-time RT-PCR for each positive sample are reported in Table 2.

## 4. Discussion

In recent decades, the epidemiology of swine coronaviruses has attracted increasing attention due to their significant economic impact and notable evolutionary potential [23]. Their high evolutionary plasticity contributes to the emergence of novel strains and increases the risk of interspecies transmission [24]. PHEV is globally distributed but generally detected at low prevalence [15].

The present study reports the first molecular detection of Porcine Hemagglutinating Encephalomyelitis Virus (PHEV) RNA in wild boars in Italy. Among the 625 nasal swabs collected during a two-year regional surveillance program, 12 samples tested positive by pan-coronavirus RT-PCR screening and were subsequently confirmed by a PHEV-specific real-time RT-PCR assay, corresponding to a prevalence of 1.9% (95% CI: 1.11–3.33%).

Although PHEV is recognized as an endemic coronavirus of domestic pigs in many countries [15,25], information regarding its circulation in free-ranging wild boars remains scarce. Most previous investigations in European wild boars have focused on enteric porcine coronaviruses or serological evidence of coronavirus exposure, whereas molecular evidence of PHEV infection is extremely limited [7,26]. While serological evidence of coronavirus exposure in Italian wild boars has previously been reported, to our knowledge this is the first molecular detection of PHEV in wild boars in Italy. Therefore, our findings expand the current knowledge of the geographic distribution of this virus and provide new epidemiological information on coronavirus circulation in wild suids.

The relatively low proportion of positive animals observed in this study suggests that active viral shedding in the upper respiratory tract was uncommon in the sampled population. However, this estimate should be interpreted cautiously because only nasal swabs were analyzed. PHEV is a neurotropic betacoronavirus that initially replicates in the upper respiratory tract before spreading through peripheral nerves to the central nervous system. Consequently, viral RNA may no longer be detectable in nasal secretions during later stages of infection, whereas neural tissues such as the trigeminal ganglia or brainstem may remain positive. Since the samples were collected within the framework of the regional wild boar surveillance program, only nasal swabs were routinely available under field conditions. Therefore, the exclusive use of this sample type may have underestimated the true occurrence of infection in the investigated population.

Another important limitation of the present study is the lack of molecular characterization of the detected viruses. Although all positive samples were confirmed using a published PHEV-specific real-time RT-PCR assay, sequencing could not be performed because the RNA extracted from the positive nasal swabs was of insufficient quality and concentration to obtain reliable sequence data. This limitation is likely related to the low viral RNA content of field-collected respiratory samples and prevented phylogenetic analyses and comparison with PHEV strains circulating in domestic pigs.

The Ct values obtained for the positive samples ranged from 22 to 36, indicating variable viral RNA loads. Samples with higher Ct values likely contained limited amounts of viral RNA, which may have contributed to the inability to obtain sequence data and further molecular characterization.

Likewise, virus isolation was not attempted because of the limited amount and quality of the available RNA, and therefore the presence of infectious virus could not be demonstrated.

For these reasons, the present study does not allow conclusions regarding the epidemiological role of wild boars as reservoirs or maintenance hosts, nor does it demonstrate transmission between wild boars and domestic pigs. Detection of viral RNA indicates only that PHEV genetic material was present in the analyzed samples at the time of collection. Further investigations combining molecular detection, virus isolation, serological surveys and genomic characterization will be necessary to clarify whether wild boars contribute to the maintenance or dissemination of PHEV under natural conditions and also if the infection could be considered active or latent.

The geographical distribution of positive animals in the provinces of Potenza and Matera is nevertheless noteworthy because these areas include a high density of pig farms, many of which operate under outdoor or semi-extensive production systems where indirect contact between domestic pigs and wildlife may occur. Although the present data do not demonstrate transmission between the two populations, they highlight the importance of integrated surveillance at the wildlife–livestock interface [27]. Within a One Health framework, monitoring wildlife populations can provide valuable information on the circulation of pathogens that may affect livestock, contributing to early detection and improved risk assessment [28].

Future studies should include broader geographical sampling, longitudinal surveillance covering different seasons, and the collection of additional tissues with higher diagnostic sensitivity, particularly neural tissues such as the trigeminal ganglia and brainstem. Whenever possible, samples suitable for whole-genome sequencing should be obtained to enable molecular characterization and comparison with strains circulating in domestic pigs. The integration of molecular, virological and serological approaches will provide a more comprehensive understanding of PHEV ecology and its circulation among wild and domestic suids.

## 5. Conclusions

This study provides the first molecular evidence of the circulation of PHEV RNA in wild boars in Italy, with 12 positive animals identified among 625 nasal swabs collected in the Basilicata Region.

Although the observed prevalence was low (1.9%), this finding expands the current knowledge of coronavirus circulation in European wild boars and highlights the value of wildlife surveillance within a One Health framework.

The results should, however, be interpreted with caution. Detection of viral RNA in nasal swabs alone does not demonstrate the presence of infectious viruses, establish wild boars as reservoirs, or indicate transmission to domestic pigs. Furthermore, the inability to perform sequencing because of the poor quality and low concentration of RNA recovered from positive samples, together with the absence of virus isolation and serological investigations, limits the epidemiological interpretation of the findings.

Future investigations integrating broader geographical surveillance, collection of tissues with greater diagnostic sensitivity, whole-genome sequencing, virus isolation and serological analyses will be essential to clarify the epidemiology of PHEV in wild boar populations and to determine the relationship between viruses circulating in wildlife and those infecting domestic pigs.

## Figures and Tables

**Table 2:** Samples tested with PHEV PCR with the relative Ct values.

ID	RNA (ng/µL)	Ct Values	Sex
13747	16.9	32	F
4971	15.8	34	F
4969	18.3	30	M
49101	20.3	22	M
15755	16.5	31	F
756138	22	25	F
756139	13.4	36	F
756147	16.7	33	F
756150	20.7	23	F
436	17	32	F
434	23.2	28	M
836	34	19	F

## Data Availability

The original contributions presented in this study are included in the article/Supplementary Material. Further inquiries can be directed to the corresponding author.

## Supplementary Materials

The following supporting information can be downloaded at: https://www.mdpi.com/article/10.3390/vetsci13080773/s1. Table S1: Distribution of sampled wild boars by collection period, municipality, sex, age class, and pan-coronavirus screening results.

## References

[1] Tack J. (2018). A scientific review of population trends and implications for management. Wild Boar (Sus scrofa) Populations in Europe.

[2] Massei G., Kindberg J., Licoppe A., Gačić D., Šprem N., Kamler J., Baubet E., Hohmann U., Monaco A., Ozoliņš J. (2014). Wild boar populations up, numbers of hunters down? A review of trends and implications for Europe. Pest Manag. Sci..

[3] Rossi A., Santi A., Barsi F., Casadei G., Di Donato A., Fontana M.C., Galletti G., Garbarino C.A., Lombardini A., Musto C. (2023). Eleven Years of Health Monitoring in Wild Boars (*Sus scrofa*) in the Emilia-Romagna Region (Italy). Animals.

[4] Cappa F., Bani L., Meriggi A. (2021). Factors affecting the crop damage by wild boar (*Sus scrofa*) and effects of population control in the Ticino and Lake Maggiore Park (North-western Italy). Mamm. Biol..

[5] Castillo-Contreras R., Carvalho J., Serrano E., Mentaberre G., Fernández-Aguilar X., Colom A., González-Crespo C., Lavín S., López-Olvera J.R. (2017). Urban wild boars prefer fragmented areas with food resources near natural corridors. Sci. Total Environ..

[6] Fredriksson-Ahomaa M. (2018). Wild Boar: A reservoir of foodborne zoonoses. Foodborne Pathog. Dis..

[7] Ferrara G., Nocera F.P., Longobardi C., Ciarcia R., Fioretti A., Damiano S., Iovane G., Pagnini U., Montagnaro S. (2022). Retrospective Serosurvey of Three Porcine Coronaviruses among the Wild Boar (*Sus scrofa*) Population in the Campania Region of Italy. J. Wildl. Dis..

[8] Meng X.J., Lindsay D.S., Sriranganathan N. (2009). Wild boars as sources for infectious diseases in livestock and humans. Philos. Trans. R. Soc. B Biol. Sci..

[9] Woo P.C.Y., Lau S.K.P., Huang Y., Yuen K.-Y. (2009). Coronavirus diversity, phylogeny and interspecies jumping. Exp. Biol. Med..

[10] Colina S.E., Serena M.S., Echeverría M.G., Metz G.E. (2021). Clinical and molecular aspects of veterinary coronaviruses. Virus Res..

[11] Li Q., Shah T., Wang B., Qu L., Wang R., Hou Y., Baloch Z., Xia X. (2023). Cross-species transmission, evolution and zoonotic potential of coronaviruses. Front. Cell. Infect. Microbiol..

[12] Dolan P.T., Whitfield Z.J., Andino R. (2018). Mapping the Evolutionary Potential of RNA Viruses. Cell Host Microbe.

[13] Ortello C., Pace L., Farina D., Manzulli V., Rondinone V., Cipolletta D., Galante D. (2026). Suidae Coronaviruses: Epidemiology, Transmission, and Molecular Diagnosis. Animals.

[14] Wang Q., Vlasova A.N., Kenney S.P., Saif L.J. (2019). Emerging and re-emerging coronaviruses in pigs. Curr. Opin. Virol..

[15] Mora-Díaz J.C., Piñeyro P.E., Houston E., Zimmerman J., Giménez-Lirola L.G. (2019). Porcine hemagglutinating encephalomyelitis virus: A review. Front. Vet. Sci..

[16] Jung K., Saif L.J. (2015). Porcine Epidemic Diarrhea Virus Infection: Etiology, Epidemiology, Pathogenesis and Immunoprophylaxis. Vet. J..

[17] Hu H., Jung K., Wang Q., Saif L.J., Vlasova A.N. (2021). Corrigendum to “Development of a one-step RT-PCR assay for detection of pancoronaviruses (α-, β-, γ-, and δ-coronaviruses) using newly designed degenerate primers for porcine and avian “fecal samples” [J. Virol. Methods 256 116–122]. J. Virol. Methods.

[18] Xiu L., Binder R.A., Alarja N.A., Kochek K., Coleman K.K., Than S.T., Bailey E.S., Bui V.N., Toh T.-H., Erdman D.D. (2020). A RT-PCR assay for the detection of coronaviruses from four genera. J. Clin. Virol..

[19] Hu X., Feng S., Shi K., Shi Y., Yin Y., Long F., Wei X., Li Z. (2023). Development of a quadruplex real-time quantitative RT-PCR for detection and differentiation of PHEV, PRV, CSFV, and JEV. Front. Vet. Sci..

[20] Rawal G., Yim-Im W., Aljets E., Halbur P.G., Zhang J., Opriessnig T. (2023). Porcine Respiratory Coronavirus (PRCV): Isolation and Characterization of a Variant PRCV from USA Pigs. Pathogens.

[21] Drzewnioková P., Festa F., Panzarin V., Lelli D., Moreno A., Zecchin B., De Benedictis P., Leopardi S. (2021). Best Molecular Tools to Investigate Coronavirus Diversity in Mammals: A Comparison. Viruses.

[22] Brown L.D., Cai T.T., DasGupta A. (2001). Interval Estimation for a Binomial Proportion. Stat. Sci..

[23] Ibrahim Y.M., Liu C., Yu Y., Yang L., Chen Q., Ma W., Werid G.M., Li S., Luo J., Gao S. (2026). Swine Enteric Coronaviruses: An Updated Overview of Epidemiology, Diagnosis, Prevention, and Control. Animals.

[24] Turlewicz-Podbielska H., Pomorska-Mól M. (2021). Porcine Coronaviruses: Overview of the State of the Art. Virol. Sin..

[25] Quiroga M.A., Cappuccio J., Piñeyro P., Basso W., Moré G., Kienast M., Schonfeld S., Cáncer J.L., Arauz S., Pintos M.E. (2008). Hemagglutinating encephalomyelitis coronavirus infection in pigs, Argentina. Emerg. Infect. Dis..

[26] Antas M., Olech M., Szczotka-Bochniarz A. (2021). Porcine Enteric Coronavirus Infections in Wild Boar in Poland—A Pilot Study. J. Vet. Res..

[27] Miller R.S., Bevins S.N., Cook G., Free R., Pepin K.M., Gidlewski T., Brown V.R. (2022). Adaptive risk-based targeted surveillance for foreign animal diseases at the wildlife-livestock interface. Transbound. Emerg. Dis..

[28] Goulet C., de Garine-Wichatitsky M., Chardonnet P., de Klerk L.M., Kock R., Muset S., Suu-Ire R., Caron A. (2024). An operational framework for wildlife health in the One Health approach. One Health.

